# Impact of cancer on cryopreserved sperm quality and fertility: A cohort study

**DOI:** 10.1002/hsr2.726

**Published:** 2022-07-20

**Authors:** David Pening, Marnie Constant, Manon Bruynbroeck, Anne Delbaere, I. Demeestere

**Affiliations:** ^1^ Department of Obstetrics and Gynecology, Fertility Clinic CUB—Erasme Hospital Brussels Belgium; ^2^ Research Laboratory on Human Reproduction Erasme Campus Université Libre de Bruxelles Brussels Belgium; ^3^ Fertility Clinic, IVF Laboratory CUB—Erasme Hospital Brussels Belgium

**Keywords:** ART, cancer, fertility preservation, semen quality

## Abstract

**Background:**

Sperm quality at cancer diagnosis is often compromised by the disease and any given gonadotoxic treatment will further diminish fertility.

**Objectives:**

Here, we aim to analyze the cryopreserved sperm quality according to the cancer types as well as the fertility outcomes.

**Methods:**

Our study included all cancer patients who cryopreserved sperm over 20 years at Erasme Hospital Brussels (from 1999 to 2019). First sperm samples from 111 hematologic, 104 testicular, 19 prostate, 28 gastrointestinal, and 16 neurological cancer patients were compared.

**Results:**

Oligozoo‐asthenozoospermia was observed in 30% of the samples, including 19.33% with severe oligozoospermia (<5 million/ml). Our results showed a significant reduction in sperm concentration among testicular cancer (*p* < 0.01). No significant differences in progressive motility, sperm volume, and number of frozen straws were observed. Significant correlations were found between sperm concentration and cancer type (*p* <0.01) as well as patients' age (*p* <0.01). Twenty‐eight cancer survivors returned for using their cryopreserved sperm (9.33%), fertilization rate was 60.5% and implantation rate was 29.6%. There was no correlation between sperm concentration and fertility outcomes.

**Conclusion:**

Our results confirm the negative impact of cancer on sperm quality without affecting assisted reproductive technology (ART) success rate, which is utterly important as a male reproductive health perspective. All cancer patients should be counselled and offered fertility preservation options as a gold standard.

**Abbreviations**
ANOVA: analysis of variance
ART: assisted reproductive technology
CPA: cryoprotective agents
DFI: DNA fragmentation index
ET: embryo transfer
ICSI: intracytoplasmic sperm injection
IUI: intrauterine insemination
IVF: in vitro fertilization
SPSS: statistical package for the social sciences
STATA: statistical software for data science
WHO: World Health Organization

## 1 INTRODUCTION

Cancer has affected 18.1 million people worldwide in 2018, leading to around 9.6 million deaths.^1^ In Belgium, cancer incidence is increasing with more than 70,000 new cases in 2018, among which 42,593 were male patients.^2^, ^3^

The most common cancers in men in reproductive age are testicular neoplasia, hematologic malignancies (including leukemia, Hodgkin and non‐Hodgkin lymphoma), colorectal cancers, and brain tumors.^2^, ^4^, ^5^

Treatments such as chemotherapy and radiotherapy have well‐known gonadotoxic effects depending on the type of drugs as well as the doses and localization of the radiation.^2^, ^6^ The cancer itself and the treatment may both impair male fertility by affecting sperm DNA and spermatogenesis, leading to temporary or permanent azoospermia.^7^, ^8^, ^9^ Moreover, evolution of the disease and response to treatment may be unpredictable and it is not excluded that a patient will eventually benefit from a second‐line gonadotoxic treatment not initially planned.^10^

Since 2018, the cryopreservation of semen is covered by the insurances up to 45 years of age in Belgium for oncological indications including solid tumors, hematopoietic or lymph node malignancies, testicular neoplasia (with or without adjuvant treatments), and all indications of hematopoietic stem cell transplantation. The sperm straws are stored for a renewable period of 10 years, unless otherwise requested by the patient himself.

Nowadays, the number of cancer survivors is increasing through the development of sensitive screening tools to detect early stage cancers and of new targeted cancer therapies.^6^, ^11^ Hence, in a society where both women and men become parents later in life,^12^ an increasing number of cancer patients do not have children or do not yet complete their family projects at the time of diagnosis. Therefore, the possibility of sperm banking before starting their potentially gonadotoxic therapies is crucial for their future quality of life as it offers them the opportunity to have children after remission.^2^, ^4^, ^5^, ^6^ This procedure is highly recommended by guidelines and considered as a gold standard to preserve fertility in adults and adolescents.^13^, ^14^

Previous studies showed sperm quality impairment before the gonadotoxic treatment due to cancer itself,^10^ focusing mainly on the testicular cancers and hematologic malignancies.^9^, ^11^ Few authors evaluated the fertility outcomes,^15^, ^16^, ^17^ emphasizing the importance of the use of frozen sperm for male cancer patients.

In this study, we aim to address these questions in a large cohort who cryopreserved sperm over a two‐decade period. The characteristics of the sperm were compared according to the type of cancer with the objective to further evaluate the correlation between the type of cancer and sperm quality. Other potential confounding factors such as smoking, alcohol consumption, andrology history, and the age of the patient at diagnosis were also considered. Fertility outcomes were analyzed as a secondary objective.

## 2 MATERIALS AND METHODS

### 2.1 Study design

This monocentric retrospective study was conducted in Brussels, at the CUB‐Erasme Hospital, Université Libre de Bruxelles. All data were collected from electronic medical records. The study was approved by the Erasme Ethical Committee (P2019/397).

The patients included in the study were male cancer patients, who performed sperm cryopreservation procedures for fertility preservation at the time of diagnosis.

Information regarding the type of cancer, sperm analysis parameters, smoking or alcohol consumption, andrology history, age, treatment already received, and history of assisted reproductive technology (ART) were collected from the medical reports. Oncological diseases were classified into different categories: hematologic malignancies (including leukemia and lymphoma), testicular, prostate, colorectal, brain, and other less common cancer types.

### 2.2 Data extraction

**Figure 1:**
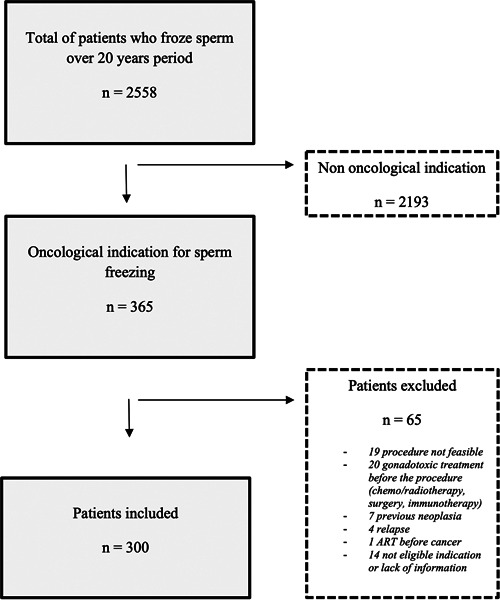
Flowchart of patients who came for sperm freezing at Erasme Hospital between January 1^st^, 1999, and June 30^th^, 2019.

A total of 2558 patients banked sperm between January 1^st^, 1999, and June 30^th^, 2019, among them 2193 were excluded as the indication was not oncological. The remaining 365 cancer patients were screened and 65 of them were excluded due to the procedure being not feasible (*n *= 19), a gonadotoxic treatment given before the procedure (*n* = 20), a previous neoplasia (*n* = 7), a relapse (*n* = 4), an ART before cancer (*n* = 1), and due to lack of information (*n* = 14). Finally, a total of 300 patients were eligible for analysis (Figure 1).

A standardized protocol was used in Erasme Andrology Lab for sperm freezing including liquefaction, semen analysis, sperm dilution with cryoprotective agents (CPA), balancing and cooling, slow freezing method in liquid nitrogen.

### 2.3 Statistical analysis

All the analyses were carried out on STATA 15 software and SPSS (version 27). Continuous data with parametric distribution are presented as mean ± SD and analyzed with the analysis of variance (ANOVA) and Turkey test. Continuous data with nonparametric distribution are presented as the median and interquartile range (IQR) and analyzed with the Kruskal–Wallis and Wilcoxon–Mann–Whiney tests. Categorical data are described by number of cases, including numerator, denominator, and percentages, and analyzed with the Fisher exact test. Regression analyses were analyzed by univariate regression analysis. A *p *value < 0.05 threshold was defined as statistically significant for all statistical tests.

## 3 RESULTS

### 3.1 Study population

he main neoplasia found were hematologic malignancies (37%) and testicular cancers (34.7%). The median age of the patient at the time of the diagnostic was 29 years (IQR 23–37), with patients diagnosed with prostate cancer being the oldest (Table 1).

**Table 1:** Characteristics of the population according to the type of neoplasia: H, T, P, GI, N, and O

**Characteristics**	Population	H	T	P	GI	N	O	*p* Value
Patients, *n* (%)	300 (100)	111 (37)	104 (34.67)	19 (6.33)	28 (9.33)	16 (5.33)	22 (7.33)	
Age, years (median, IQR)	29 (23–37)	28 (22–35.5)	27 (23–31.25)	50 (46.5–54)	36 (26.65–38)	27 (20–34.75)	37 (24.5–45.75)	**<0.01** a
Tobacco, *n* (%)								0.564^b^
Smoker	91 (30.33)	33 (29.73)	36 (34.62)	6 (31.58)	4 (14.29)	4 (25.00)	8 (36.36)	
Nonsmoker	165 (55.00)	61 (54.95)	55 (52.88)	11 (57.89)	18 (64.29)	11 (68.75)	9 (40.91)	
Unknown	44 (14.67)	17 (15.32)	13 (12.50)	2 (10.53)	6 (21.43)	1 (6.25)	5 (22.73)	
Alcohol consumption, *n* (%)								0.535^b^
Yes, daily	22 (7.33)	7 (6.31)	7 (6.73)	5 (26.32)	2 (7.14)	1 (6.25)	0 (0.00)	
Yes, occasionally	55 (18.33)	20 (18.02)	25 (24.04)	2 (10.53)	3 (10.71)	2 (12.50)	3 (13.64)	
Yes, unknown frequency	4 (1.33)	2 (1.80)	2 (1.92)	0 (0.00)	0 (0.00)	0 (0.00)	0 (0.00)	
No	175 (58.33)	65 (58.56)	57 (54.81)	10 (52.63)	17 (60.71)	12 (75.00)	14 (63.64)	
Unknown	44 (14.67)	17 (15.32)	13 (12.50)	2 (10.53)	6 (21.43)	1 (6.25)	5 (22.73)	
Andrological history, *n* (%)								0.672^b^
Yes	27 (9.00)	11 (9.10)	11 (10.58)	1 (5.26)	0 (0.00)	1 (6.25)	3 (13.64)	
No	142 (47.33)	52 (46.85)	48 (46.15)	7 (36.84)	15 (53.57)	9 (56.25)	11 (50.00)	
Unknown	131 (43.67)	48 (43.24)	45 (43.27)	11 (57.89)	13 (46.43)	6 (37.5)	8 (36.36)	
Proven fertility, *n* (%)								0.266^b^
Yes	64 (21.33)	21 (18.92)	20 (19.23)	5 (26.32)	7 (25.00)	3 (18.75)	8 (36.36)	
No	115 (38.33)	43 (38.74)	49 (47.12)	4 (21.05)	9 (32.14)	3 (18.75)	7 (31.82)	
Unknown	121 (40.33)	47 (42.34)	35 (33.65)	10 (52.63)	12 (42.86)	10 (62.50)	7 (31.82)	

*Note*: Variables are presented by median surrounded IQRs or *n* and percentage (%). *p* Value considered significant when <0.05.

Abbreviations: GI, gastrointestinal; H, hematologic; IQR, interquartile interval; N, neurological; O, others include 3 teratoma, 1 penile cancer, 1 tongue cancer, 2 osteosarcoma, 4 multiple myeloma, 1 bladder cancer, 4 lung cancers, 6 head and neck cancers; P, prostate; T, testicular.

^a^ Kruskal–Wallis.

^b^ Fisher's exact test without the unknown.

No significant differences in tobacco and alcohol consumption, andrology history, and pre‐cancer paternity were observed between the categories (Table 1). Twenty‐one percent of the patients had at least one child at the time of the diagnosis, however, the information was missing for one‐third of the patients (121 patients).

### 3.2 Sperm parameters

Sperm concentration was significantly lower in patients with testicular cancer compared to the other cancer groups (hematologic, prostate, and colorectal neoplasia). No statistical differences were observed between the studied groups for the volume of the ejaculate, the sperm progressive motility, and the number of frozen straws per sample. Regression analysis also confirmed a lower semen concentration in the testicular cancer group compared to the hematologic malignancies group (Table 2).

**Table 2:** Characteristics of the sperm samples according to the type of neoplasia

Characteristics	H	T	P	GI	N	O	*p* Value^a^
Volume, ml							
Median (IQR)	2.5 (1.5–3.9)	3 (2–4.1)	2 (1.85–2.45)	3 (2.02–4.25)	2.65 (1.85–3.62)	2.75 (2.02–3.42)	0.175
	(*n *= 111)	(*n* = 104)	(*n* = 19)	(*n* = 28)	(*n* = 16)	(*n* = 22)	
Concentration, million/ml							
Median (IQR)	42 (14.65–84.5)	13.85 (4.75–43.75)	55.55 (21.9–89.5)	48.8 (18.52–116.25)	34.75 (17.32–152)	34.5 (16.5–78.75)	**<0.01**
	(*n* = 111)	(*n* = 104)	(*n *= 19)	(*n *= 28)	(*n* = 16)	(*n* = 22)	
Progressive motility,^b^ %							
Median (IQR)	41 (27.25–52)	51 (38–62.75)	31 (23‐60)	43 (25.75–57.25)	28 (16.5–48.5)	32 (22–52.5)	0.637
	(*n* = 102)	(*n* = 86)	(*n *= 17)	(*n* = 28)	(*n* = 15)	(*n* = 19)	
Number of frozen straws, *n*							
Median (IQR)	8 (3.5–12)	8 (4–12)	6 (4–8)	9 (5–13)	9 (5.75–10.25)	8 (6.25–11)	0.848
	(*n* = 111)	(*n* = 104)	(*n* = 19)	(*n* = 28)	(*n *= 16)	(*n* = 22)	

*Note*: Variables are presented by median surrounded IQRs and number of straws (*n*).

Abbreviations: GI, gastrointestinal; H, hematologic; IQR, interquartile interval; N, neurological; O, others include 3 teratoma, 1 penile cancer, 1 tongue cancer, 2 osteosarcoma, 4 multiple myeloma, 1 bladder cancer, 4 lung cancers, 6 head and neck cancers; P, prostate; T, testicular.

^a^ *p* Values are calculated with Kruskal–Wallis's test*,* significant *p* values < 0.05*.*

^b^ Information is not available for all patients.

**Figure 2:**
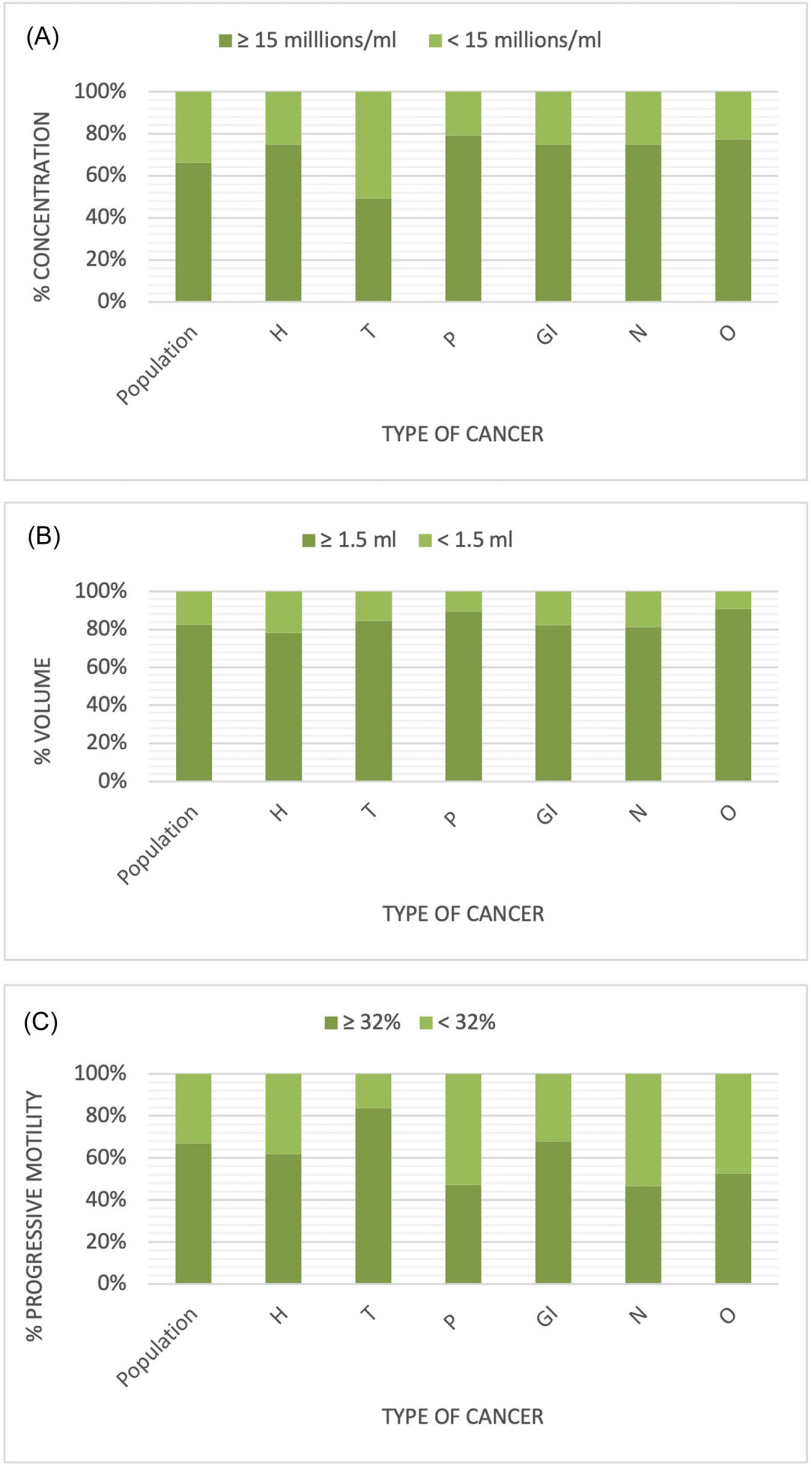
Distribution of concentration in millions per ml (A), volume in ml (B), and progressive motility (C) of the studied population and six cancer groups sperm analysis, according to the WHO 2010 reference values.

Based on the WHO 2010 criteria, more than 30% of the collected samples are considered as oligozoo‐asthenozoospermia, including 58 patients (19.33%) with severe oligospermia (<5 million/ml). Among them, half had testicular neoplasia (Figure 2). No statistical differences of sperm parameters were observed between Hodgkin lymphoma, non‐Hodgkin lymphoma, and leukemia patients with hematologic malignancies (Supporting Information).

### 3.3 Fertility outcomes

Over 20 years, 28 patients returned for using their frozen sperm after a median of 4.25 years. A total of 85 ART cycles were performed including 38 intrauterine insemination (IUI), 1 in vitro fertilization (IVF), and 46 intracytoplasmic sperm injection (ICSI) cycles. A total of 91 embryos were transferred: 52 were fresh embryo transfer (ET) and 39 were frozen ET. The overall fertilization rate (FR) and implantation rate (IR) reached 60.5% and 29.6%, respectively.

The characteristics of the cohort including maternal age, sperm parameters, indications, and fertility outcomes are provided in Table 3 and Supporting Information. No correlation was observed between sperm concentration and pregnancy success. No significant differences in the sperm parameters were observed between the sperm of patients who achieved pregnancy and those who failed.

**Table 3:** Reproductive outcomes of 28 patients stratified by cancer types

Characteristics	Population	H	T	O	*p* Value^a^
Number of patients,* n*	28	8	11	9	
Male age, years	35.5 (32.5–45)	33.5 (32.5–44)	33 (31–36)	49 (40–51)	**0.004**
Female age, years	32.5 (30–36)	33 (30–38)	31 (28–33)	35 (32–37)	0.12
Delay after cancer, years	4.25 (0.16–13.1)	2.76 (0.47–4.98)	6.08 (1.77–13.1)	2.78 (0.16–6.21)	
Type of ART using thawed sperm					
IUI,* n* cycles	38	16	10	12	
IVF,* n* cycles	1	0	0	1	
ICSI, *n* cycles	46	10	26	10	
Number of oocytes					
Inseminated (no. MII)	296	64	161	71	0.40
Fertilized (no. 2 PN)	179	33	103	43	0.29
No. fresh embryo transfer	52	13	28	11	0.43
No. frozen embryo transfer	39	5	20	14	0.53
Fertilization rate (%)	60.47	51.56	63.98	60.56	0.72
Implantation rate (%)	29.61	20.00	36.25	22.50	0.42
Live birth rate (%)	52.20	57.14	64.29	45.71	0.84

*Note*: Variables are presented by median surrounded IQRs, or *n* and %.

Abbreviations: FR, fertilization rate defined as no. oocytes with 2 PN divided by no. MII oocytes injected (for ICSI) or divided by no. COCs inseminated (for IVF); H, hematologic; ICSI, intracytoplasmic sperm injection; IQR, interquartile interval; IVF, in vitro fertilization; IR, implantation rate defined as no. gestational sacs seen on ultrasound divided by no. embryos transferred; IUI, intrauterine insemination; LBR, live birth rate defined as one infant born after 24 weeks of gestation after fresh embryo transfer or after cryopreserved embryo transfer; O, other details: 3 prostate cancer, 2 gastrointestinal cancer, 1 brain cancer, 1 bladder cancer, 1 multiple myeloma, 1 neck cancer; PN, pronuclei stage; T, testicular.

^a^ *p* Values are calculated with Kruskal–Wallis's Test, significant *p* value < 0.05.

## 4 DISCUSSION

Testicular cancer and hematologic (leukemia and lymphoma) malignancies are the main indications of sperm freezing in our population, which is in line with the literature.^10^, ^15^, ^18^ Overall, more than 30% of patients were oligozoospermic at the time of diagnosis. Almost 20% of them had severe oligozoospermia according to WHO 2010 semen analysis criteria (<5 million/ml), half of whom being testicular cancers.^19^

We confirmed that sperm concentration was significantly lower among testicular cancer patients compared to other cancer types (hematologic including both leukemia and lymphoma, prostate, neurological and colorectal cancers). A hypothesis being the direct cancer effect on spermatogenesis impairment and the indirect effect related to endocrine and systemic disturbances.^20^ It is worth noting that the decline in sperm quality among testicular cancer patients is often associated with local inflammation, a history of cryptorchidism, and/or hypospadias referred as “testicular dysgenesis syndrome.”^21^

A study by Song et al. also showed a statistically significant reduced sperm concentration in testicular cancer patients compared to other neoplasia but 55.6% of the testicular cancer patients had an orchidectomy before sperm cryopreservation.^22^ In our data extraction, the information regarding orchidectomy was not always available. Usually, sperm cryopreservation was performed before the surgery. Other studies reported a diminished sperm concentration in testicular cancer patients compared to hematologic malignancies,^11^ or to other cancers without significant changes in motility and volume.^23^, ^24^ A large case–control study in 4480 male patients also showed that human sperm concentration was four‐fold lower among testicular cancer (19.6 million/ml) than in the normal population (82.4 million/ml).^25^ The median concentration of spermatozoa was even lower in our population (13.85 million/ml; IQR 4.75–43).

Among hematologic malignancies, we did not observe any significant differences between the subgroups (leukemia, Hodgkin, and non‐Hodgkin lymphoma) in terms of sperm parameters. A study by Caponecchia et al. showed a lower sperm concentration in both testicular cancer and Hodgkin lymphoma compared to the native disease‐free population.^11^ A lower concentration and motility were also reported in leukemia patients compared to other cancer types.^18^, ^22^ Finally, a large case–control study reported only 36.7% normospermic samples in leukemia patients.^25^

For brain neoplasia, similar sperm count and concentration were reported compared to the control population, but with lower motility (30%) and a diminished normal morphology rate.^25^ For our patients, sperm progressive motility (28%) was similar as previously reported but not significantly different compared to other diseases.

No significant relationship between sperm concentration and habits such as smoking, alcohol consumption, or andrology history were observed in our study. This might be because most of our patients were non‐smoker (55%) and did not drink alcohol for 58.3% of them. With regards to alcohol consumption units, this data remains unknown for 14.7% of our patients but we do know that heavy smoking and alcohol abuse are both deleterious for sperm quality.^26^, ^27^ Nevertheless, these parameters should be taken into account in an andrology assessment as they induce oxidative stress and DNA damage.^28^, ^29^, ^30^

Age was also considered as an important parameter as a meta‐analysis showed a trend of a lower sperm concentration with increasing paternal age, due to endocrine disruptors and accumulated oxidative stresses.^31^ A recent study from 2021, showed a negative correlation between age and sperm volume, motility and morphology; and a positive correlation between DNA fragmentation index and sperm concentration, but results lack information on male cancer history.^32^ In our study, a significant correlation was found between sperm concentration and cancer type (*p* < 0.01) as well as patients' age (*p* < 0.01) among the testicular group being the lowest and youngest, respectively. Another study by Johnson et al. showed that cancer patients are younger compared to infertile patients (29 vs. 36.4 years old, *p* < 0.001), although they are at higher risk of cryopreservation failure due to azoospermia (5% vs. 1%, *p* = 0.007).^18^

Most importantly, we have evaluated the fertility outcomes in our study. In total, 64 patients (21.33%) already had one child before cryopreservation. Although the data were lacking for a third of them, it highlights the importance of referring cancer patients for sperm cryopreservation before any potential gonadotoxic treatment irrespective of their previous fertility history.^8^, ^9^, ^10^, ^17^

In our cohort, only 28 patients (9.33%) returned to attempt conception using assisted reproduction technology ART (38 IUI cycles, 1 IVF, and 46 ICSI cycles) after a median time of 4.25 years. This is in line with the literature, with the usage rate varying from 7.5% to 10.3%.^7^, ^24^, ^33^ Among the 38 IUI cycles with frozen‐thawed sperm, five patients achieved a pregnancy, and four healthy babies were born. After IUI failure, seven patients went for an ICSI procedure which succeeded in pregnancy and delivery for five of them. With regards to age among cancer survivors who returned for ART, we found a statistically significant difference between the groups for male age, the testicular cancer group being the youngest compared to the other group (*p* = 0.004).

A total of 91 embryos were transferred: 52 were fresh ET and 39 were frozen ET, overall FR was 60.5% and IR was 29.6%. We obtained comparable IR of 36.2% and 20% for testicular cancer and hematologic group respectively as found in the literature.^15^ Another study on the clinical outcomes of ART included 34 ICSI and 11 frozen ET reported IR of 22% and 25% for day 3 and day 5 ET.^17^

It should be noted that among the patients who failed to conceive, two patients were still under ART treatment, two couples had separated during the ART procedure, one patient refused IVF, one patient changed to another hospital, and one patient had cancer recurrence before being able to have a child.

Finally, a 2021 study compared thawed sperm versus fresh sperm used for IVF/ICSI in cancer patients. The pregnancy rates and live birth rates were similar in the two groups.^16^ In our study, we did not observe any correlation between the frozen sperm concentration and fertility outcomes. These results are important to promote sperm cryopreservation before oncological treatment and should be discussed during fertility preservation counselling.

Our limitations are related to the retrospective design of the study, that some data were missing, and for 19 cancer patients their sperm sample did not enable a freezing procedure (extremely poor sperm sample without enough motile sperm for freezing, and/or azoospermia) but the azoospermic rate was not evaluated in the present study as they were not always identifiable in the patient's selection.

## 5 CONCLUSION

Sperm quality at cancer diagnosis is often compromised by the disease and any given gonadotoxic treatment will further diminish fertility. We analyzed sperm quality in a large cancer patients' cohort and included their fertility outcomes. The main findings were the significant reduction in sperm quality in testicular cancer patients, with a significant correlation found between sperm concentration and cancer type as well as patients' age. However, we showed no correlation between sperm concentration and fertility outcomes. Our results further confirm the negative impact of cancer on sperm quality without affecting ART success rate, which is utterly important as a male reproductive health perspective for cancer survivors. All cancer patients should be counselled and offered fertility preservation options as a gold standard.

## AUTHOR CONTRIBUTIONS

**David Pening:** Conceptualization; formal analysis; methodology; writing original draft; writing review and editing. **Marnie Constant:** Conceptualization; data curation; formal analysis; resources; writing original draft. **Manon Bruynbroeck:** Project administration; validation. **Anne Delbaere:** Writing review and editing. **Isabelle Demeestere:** Supervision; writing original draft; writing review and editing. All authors have read and approved the final version of the manuscript. David Pening has full access to all of the data in this study and takes complete responsibility for the integrity of the data and the accuracy of the data analysis.

## CONFLICTS OF INTEREST

David Pening, Marnie Constant, and Manon Bruynbroeck have no conflicts of interest to declare. Anne Delbaere acted as advisory board and received grants or lecture fees from Merck, Gedeon‐Richter, Ferring Pharmaceuticals, and OVVI Diagnostics and travel grants from Merck, Theramex, and Ferring Pharmaceuticals unrelated to the present study. Isabelle Demeestere has acted as a scientific advisory board member and received grant from ROCHE, speaker's fees from Novartis and ROCHE, and travel grants from Theramex and Ferring, outside the submitted work.

## TRANSPARENCY STATEMENT

David Pening affirms that this manuscript is an honest, accurate, and transparent account of the study being reported; that no important aspects of the study have been omitted; and that any discrepancies from the study as planned (and, if relevant, registered) have been explained.

## ETHICS STATEMENT

The study was approved by the Erasme Ethical Committee (P2019/397).

## Supporting information

Supporting information.

## Data Availability

The data are available on request from the corresponding author.

## References

[1] Bray F , Ferlay J , Soerjomataram I , Siegel RL , Torre LA , Jemal A .Global cancer statistics 2018: GLOBOCAN estimates of incidence and mortality worldwide for 36 cancers in 185 countries. CA Cancer J Clin. 2018;68(6):394‐424. 30207593 10.3322/caac.21492

[2] Coccia PF , Pappo AS , Beaupin L , et al.Adolescent and young adult oncology, version 2.2018, NCCN clinical practice guidelines in oncology. J Natl Compr Canc Netw. 2018;16(1):66‐97. 29295883 10.6004/jnccn.2018.0001

[3] Belgian Cancer Registry. Tableaux sur base annuelle*—Belgian Cancer Registry*; 2021.

[4] Fidler MM , Gupta S , Soerjomataram I , Ferlay J , Steliarova‐Foucher E , Bray F . Cancer incidence and mortality among young adults aged 20‐39 years worldwide in 2012: a population‐based study.Lancet Oncol. 2017;18(12):1579‐1589. 29111259 10.1016/S1470-2045(17)30677-0

[5] International Agency for Research on Cancer WHO . *Cancer Today*; 2021.

[6] Lambertini M , Peccatori FA , Demeestere I , et al. Fertility preservation and post‐treatment pregnancies in post‐pubertal cancer patients: ESMO Clinical Practice Guidelines†. Ann Oncol. 2020;31(12):1664‐1678. 32976936 10.1016/j.annonc.2020.09.006

[7] Ferrari S , Paffoni A , Reschini M , et al. Variables affecting long‐term usage rate of sperm samples cryopreserved for fertility preservation in cancer patients.Andrology. 2021;9(1):204‐211. 32814364 10.1111/andr.12894

[8] Kawai K , Nishiyama H . Preservation of fertility of adult male cancer patients treated with chemotherapy.Int J Clin Oncol. 2019;24(1):34‐40. 30353257 10.1007/s10147-018-1333-0

[9] Bahadur G , Ozturk O , Muneer A , et al. Semen quality before and after gonadotoxic treatment.Hum Reprod. 2005;20(3):774‐781. 15689346 10.1093/humrep/deh671

[10] Shankara‐Narayana N , Di Pierro I , Fennell C , et al. Sperm cryopreservation prior to gonadotoxic treatment: experience of a single academic centre over 4 decades. Hum Reprod. 2019;34(5):795‐803. 30951144 10.1093/humrep/dez026

[11] Caponecchia L , Cimino G , Sacchetto R , et al. Do malignant diseases affect semen quality? Sperm parameters of men with cancers. Andrologia. 2016;48(3):333‐340. 26173956 10.1111/and.12451

[12] Khandwala YS , Zhang CA , Lu Y , Eisenberg ML . The age of fathers in the USA is rising: an analysis of 168 867 480 births from 1972 to 2015. Hum Reprod. 2017;32(10):2110‐2116. 28938735 10.1093/humrep/dex267

[13] Demeestere I , Racape J , Dechene J , et al. Gonadal function recovery in patients with advanced hodgkin lymphoma treated with a PET‐adapted regimen: prospective analysis of a randomized phase III trial (AHL2011). J Clin Oncol. 2021;39(29):3251‐3260. 34156881 10.1200/JCO.21.00068

[14] Mulder RL , Font‐Gonzalez A , Green DM , et al. Fertility preservation for male patients with childhood, adolescent, and young adult cancer: recommendations from the PanCareLIFE consortium and the international late effects of childhood cancer guideline harmonization group. Lancet Oncol. 2021;22(2):e57‐e67. 33539754 10.1016/S1470-2045(20)30582-9

[15] Depalo R , Falagario D , Masciandaro P , et al. Fertility preservation in males with cancer: 16‐year monocentric experience of sperm banking and post‐thaw reproductive outcomes. Ther Adv Med Oncol. 2016;8(6):412‐420. 27800030 10.1177/1758834016665078 PMC5066546

[16] Papler TB , Vrtacnik‐Bokal E , Drobnic S , Stimpfel M . The outcome of IVF/ICSI cycles in male cancer patients: retrospective analysis of procedures from 2004 to 2018. Radiol Oncol. 2021;55(2):221‐228. 33675201 10.2478/raon-2021-0011 PMC8042825

[17] Stigliani S , Massarotti C , De Leo C , et al. Fifteen year regional center experience in sperm banking for cancer patients: use and reproductive outcomes in survivors. Cancers. 2021;13(1):116. 10.3390/cancers13010116 PMC7796110 33401381

[18] Johnson MD , Cooper AR , Jungheim ES , Lanzendorf SE , Odem RR , Ratts VS . Sperm banking for fertility preservation: a 20‐year experience. Eur J Obstet Gynaecol Reprod Biol. 2013;170(1):177‐182. 10.1016/j.ejogrb.2013.06.021 23870186

[19] Cooper TG , Noonan E , von Eckardstein S , et al. World Health Organization reference values for human semen characteristics. Hum Reprod Update. 2010;16(3):231‐245. 19934213 10.1093/humupd/dmp048

[20] Bujan L , Walschaerts M , Moinard N , et al. Impact of chemotherapy and radiotherapy for testicular germ cell tumors on spermatogenesis and sperm DNA: a multicenter prospective study from the CECOS network. Fertil Steril. 2013;100(3):673‐680. 23755953 10.1016/j.fertnstert.2013.05.018

[21] Skakkebæk NE , Rajpert‐De Meyts E , Main KM . Testicular dysgenesis syndrome: an increasingly common developmental disorder with environmental aspects: opinion. Hum Reprod. 2001;16(5):972‐978. 11331648 10.1093/humrep/16.5.972

[22] Song S‐H , Kim DK , Sung SY , et al. Long‐term experience of sperm cryopreservation in cancer patients in a single fertility center. World J Mens Health. 2019;37(2):219‐225. 30588786 10.5534/wjmh.180061 PMC6479079

[23] McDowell S , Harrison K , Kroon B , Ford E , Yazdani A . Sperm DNA fragmentation in men with malignancy. Fertil Steril. 2013;99(7):1862‐1866. 23481280 10.1016/j.fertnstert.2013.02.015

[24] Botchan A , Karpol S , Lehavi O , et al. Preservation of sperm of cancer patients: extent of use and pregnancy outcome in a tertiary infertility center. Asian J Androl. 2013;15(3):382‐386. 23524529 10.1038/aja.2013.3 PMC3739646

[25] Auger J , Sermondade N , Eustache F . Semen quality of 4480 young cancer and systemic disease patients: baseline data and clinical considerations. Basic Clin Androl. 2016;26:3. 26893905 10.1186/s12610-016-0031-x PMC4758099

[26] Amor H , Hammadeh ME , Mohd I , Jankowski PM . Impact of heavy alcohol consumption and cigarette smoking on sperm DNA integrity. Andrologia. 2022;54(7):e14434. 35484935 10.1111/and.14434

[27] Penzias A , Bendikson K , Butts S , et al. Smoking and infertility: a committee opinion. Fertil Steril. 2018;110(4):611‐618. 30196946 10.1016/j.fertnstert.2018.06.016

[28] Aboulmaouahib S , Madkour A , Kaarouch I , et al. Impact of alcohol and cigarette smoking consumption in male fertility potential: looks at lipid peroxidation, enzymatic antioxidant activities and sperm DNA damage. Andrologia . 2018;50(3). Published online November 21, 2021. 10.1111/and.12926 29164649

[29] Mostafa RM , Nasrallah YS , Hassan MM , Farrag AF , Majzoub A , Agarwal A . The effect of cigarette smoking on human seminal parameters, sperm chromatin structure and condensation. Andrologia. 2018;50(3):e12910. 10.1111/and.12910 29124782

[30] Sharma R , Harlev A , Agarwal A , Esteves SC . Cigarette smoking and semen quality: a new meta‐analysis examining the effect of the 2010 world health organization laboratory methods for the examination of human semen. Eur Urol. 2016;70(4):635‐645. 27113031 10.1016/j.eururo.2016.04.010

[31] Johnson SL , Dunleavy J , Gemmell NJ , Nakagawa S . Consistent age‐dependent declines in human semen quality: a systematic review and meta‐analysis. Ageing Res Rev. 2015;19:22‐33. 25462195 10.1016/j.arr.2014.10.007

[32] Gao J , Yuan R , Yang S , et al. Age‐related changes in human conventional semen parameters and sperm chromatin structure assay‐defined sperm DNA/chromatin integrity. Reprod Biomed Online. 2021;42(5):973‐982. 33785305 10.1016/j.rbmo.2021.02.006

[33] van Casteren NJ , van Santbrink EJP , van Inzen W , Romijn JC , Dohle GR . Use rate and assisted reproduction technologies outcome of cryopreserved semen from 629 cancer patients. Fertil Steril. 2008;90(6):2245‐2250. 18191846 10.1016/j.fertnstert.2007.10.055

