# Supplementary material for: Impact of cancer on cryopreserved sperm quality and fertility: A cohort study
Source: Health Sci Rep. 2022 Jul 20;5(4):e726. doi: 10.1002/hsr2.726 (PMC9297376; doi:10.1002/hsr2.726)
Supplement: Supplementary file 1 — Supporting information. [file HSR2-5-e726-s001.docx]

| **Table 4:** **Characteristics of sperm samples among hematologic malignancies: Hodgkin Lymphoma, Non-Hodgkin Lymphoma and Leukemia.** | | | | |
| --- | --- | --- | --- | --- |
| Characteristics | HL | NHL | L | p value^#^ |
|  |  |  |  |  |
| **Volume – ml** |  |  |  |  |
| Median (IQR) | 2.7 (1.6-3.77)  (n=58) | 2.95 (1.47-4)  (n=28) | 2 (1.55-3.55)  (n=19) | 0.665 |
|  |  |  |  |  |
| **Concentration – millions/ml** |  |  |  |  |
| Median (IQR) | 37.5 (10.7-59.5)  (n=58) | 41 (14.47-76.75)  (n=28) | 78 (17.2-111)  (n=19) | 0.209 |
|  |  |  |  |  |
| **Progressive mobility**^##^ – % |  |  |  |  |
| Median (IQR) | 46.5 (37.25-57.75)  (n=50) | 28 (15-46.5)  (n=27) | 41 (30.25-48)  (n=19) | 0.552 |
|  |  |  |  |  |
| **Number of frozen straws – n** |  |  |  |  |
| Median (IQR) | 7 (3-11) | 7.5 (3-13.25) | 8 (6-12) | 0.114 |
|  |  |  |  |  |
| HL = Hodgkin Lymphoma, NHL = Non-Hodgkin Lymphoma, L= Leukemia  Variables are presented by median surrounded interquartile intervals (IQR) and number of straws (n).  *^#^* P *value*s are calculated with *Kruskal-Wallis*’s Test*,* significant *p-value <0.05*  *^##^* Information is not available for all patients  Note that 6 patients are not included because the sub-type of lymphoma was not described. | | | | |

| **Table 5: Characteristics of sperm frozen straws used in assisted reproduction technologies (ART)** | | | | | | | |
| --- | --- | --- | --- | --- | --- | --- | --- |
| Characteristics | Population | H | T | P | GI | N | O |
|  |  |  |  |  |  |  |  |
| **Number of patients *– n*** | 28 | 8 | 11 | 3 | 2 | 1 | 3 |
|  |  |  |  |  |  |  |  |
| **Volume *– ml*** |  |  |  |  |  |  |  |
| Median *(Min-Max)* | 3.1 (0.5-10)  (n=28) | 3.7 (1.2-8.5)  (n=8) | 3.7 (1-10)  (n=11) | 2 (2-2.3)  (n=3) | 4.8 (3.3-6.3)  (n=2) | 0.5  (n=1) | 3 (2.1-4.1)  (n=3) |
|  |  |  |  |  |  |  |  |
| **Concentration *– million/ml*** |  |  |  |  |  |  |  |
| Median *(Min-Max)* | 37.2 (0.1-194)  (n=28) | 105 (2.1-194) (n=8) | 12 (0.1-166)  (n=11) | 55.5 (23.2-104)  (n=3) | 44 (40.4-47.6)  (n=2) | 167  (n=1) | 31 (18-34)  (n=3) |
|  |  |  |  |  |  |  |  |
| **Progressive motility^##^ *– %*** |  |  |  |  |  |  |  |
| Median *(Min-Max)* | 48 (8-72)  (n=27) | 50.5 (34-71)  (n=8) | 46 (22-72)  (n=10) | 23 (10-49)  (n=3) | 30 (26-34)  (n=2) | 49  (n=1) | 46 (8-60)  (n=3) |
|  |  |  |  |  |  |  |  |
| H = Hematologic, T = Testicular, P = Prostate, GI = Gastrointestinal, N = Neurological, O = Other details: 1 bladder cancer, 1 multiple myeloma, 1 neck cancer  Variables are presented by median surrounded interquartile intervals (IQR) or minimum-maximum (min-max), or number (n) and percentage (%)  *^##^* Information is not available for all patients | | | | | | | |
